# Systemic Thrombolysis for Treatment of Postpartum Saddle Embolism Complicated by Postpartum Hemorrhage: A Case Report and Brief Literature Review

**DOI:** 10.1155/2021/5553296

**Published:** 2021-06-29

**Authors:** Kathy Mostajeran, Hillary Boswell, Ziad Haidar

**Affiliations:** Gulf Coast Educational Consortium/HCA Houston Healthcare West, 12141 Richmond Ave. Houston, TX 77082, USA

## Abstract

Venous thromboembolic events (VTE), specifically pulmonary embolisms, account for a significant portion of maternal morbidity and mortality. Due to the procoagulant physiological changes that occur, pregnancy and the postpartum period are known risk factors for thromboembolic events. The risk is greatest during the first-week postpartum and remains elevated for up to six weeks as compared to the general population. Treatment guidelines regarding the use of thrombolytics for massive pulmonary embolism occurring in pregnancy and the postpartum are not well established. In nonpregnant populations, thrombolytic agents are well known to decrease the mortality in the setting of a massive pulmonary embolism. However, in the absence of management guidelines, thrombolysis in pregnancy remains guided by case reports and case series. We present a case of a massive pulmonary embolism (PE) causing hemodynamic instability during the postpartum period treated with tissue plasminogen activator (tPA). The case was complicated by delayed postpartum hemorrhage successfully managed with the uterotonic methylergometrine. The patient was started on oral anticoagulation and continued for six months without recurrent VTE. Our case demonstrates a rare occurrence of a saddle embolism after a vaginal delivery within the first postpartum week which was successfully managed with the use of systemic thrombolysis and minimal intervention to manage the iatrogenic delayed postpartum hemorrhage. To the authors' knowledge, no other similar case report exists. This case highlights the need to develop guidelines for the use of thrombolysis in mothers who present with massive pulmonary embolus and a noninvasive means to manage adverse bleeding events in the puerperium.

## 1. Introduction

According to the Center of Disease Control Pregnancy Mortality Surveillance System, pulmonary embolism accounted for approximately 9.4% (*n* = 281) of the maternal deaths between 2011 and 2015 in the United States [1]. The rate of venous thromboembolism (VTE) is approximately 1.72 per 1,000 deliveries with 1.1 deaths per 100,000 [2]. While pulmonary embolism is a rare occurrence in pregnancy and the puerperium, it can have devastating consequences when not recognized early and treated aggressively.

Pregnancy and the postpartum period exemplify Virchow's triad of initiating factors for venous thrombosis, namely, hypercoagulability, increased venous stasis, and vascular damage. Pomp et al. published data findings of thromboembolic events that occurred during pregnancy and the postpartum period in the MEGA study [3]. Researchers discovered an increased relative risk of thromboembolic events during the first 6-week postpartum, with the highest risk for occurrence during the second postpartum week [3]. When compared to the third trimester, the risk of pulmonary embolism was greater during the postpartum period [3].

Pregnancy and the postpartum period are considered a relative contraindication for the use of thrombolytics, due to the lack of controlled studies and the fear of life-threatening hemorrhage [4]. Relative contraindications to the use of thrombolytics include recent surgery and active bleeding, common scenarios in the postpartum state [4]. Published research is limited to case studies and systematic reviews of published case studies which highlight the beneficial effects of thrombolysis in treatment of pulmonary embolism in pregnancy. Fewer case studies exist to evaluate the use of thrombolytic treatment in the postpartum period. Systematic reviews of case studies have revealed that in the setting of massive pulmonary embolism in the postpartum period, the maternal survival rate is 86.4%; however, the bleeding risk is approximately 58.3% [5].

We present a case of a 30-year-old female who was diagnosed with a saddle pulmonary embolism and acute hemodynamic decompensation six days after a normal spontaneous vaginal delivery. She was treated successfully with systemic thrombolysis with recovery of right ventricular function. The case was complicated by a delayed postpartum hemorrhage managed with only uterotonics. The patient was successfully transitioned to Rivaroxaban and treated for six-month duration without recurrence of VTE.

## 2. Case Report

**Figure 1:**
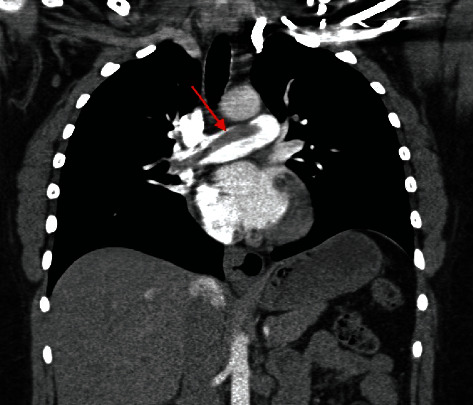
Computed tomography angiography coronal view demonstrated a large saddle embolism as shown by the red arrow.

**Figure 2:**
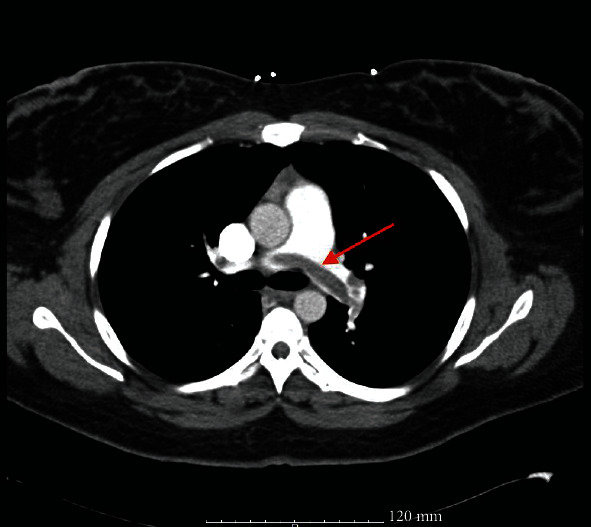
Computed tomography of the chest, transverse view, demonstrated a large saddle embolus as indicated by the red arrow.

A 30-year gravida 2 para 2002 female presented to an outside facility's emergency department with a chief complaint of syncopal episodes on postpartum day number 6 after a normal spontaneous vaginal delivery and a right mediolateral episiotomy. Her past medical history was significant for pregestational type 2 diabetes mellitus, reportedly well controlled with metformin, with a BMI of 40 kg/m^2^. She reported that she had been active at home with no history of recent prolonged travel or bed rest during the postpartum period. The patient's obstetrical history was previously uncomplicated. However, delayed placenta delivery and a right mediolateral episiotomy during her most recent vaginal delivery were reported. She had no personal or family history of thromboembolic events. She had one syncopal episode the night prior to her presentation followed by a second one the next morning while washing her face. After the second episode, she sought treatment at the closest emergency department and reported having had two additional syncopal episodes, one of which occurred in the emergency department. Shortly after arrival, the patient became hypoxic and hypotensive, with blood pressure measuring 88/50 and 88% oxygen saturation. She developed shortness of breath, and a stat computed tomography angiography of the chest demonstrated a saddle pulmonary embolus as seen in Figures 1 and 2. The decision was made to begin the infusion protocol of 100 mg tissue plasminogen activator (tPA) over the course of two hours prior to transfer to our hospital for a higher level of care in the intensive care unit.

The patient reported that prior to receiving the tPA infusion, her lochia was minimal but had increased since the tPA infusion. At the outlying facility, the patient's hemoglobin was 12.7 g/dL upon presentation. Upon arrival at our hospital, the patient's initial hemoglobin and hematocrit were 10.7 g/dL and 33.5%, respectively. The patient complained of shortness of breath; however, she remained hemodynamically stable and maintained an oxygen saturation of 100% while receiving 2 L of oxygen via nasal cannula. Her shortness of breath improved shortly after admission to the intensive care unit. The tPA infusion was completed at our hospital. An activated partial thromboplastin time (aPTT) at baseline was 42 seconds, and the patient was started on intravenous unfractionated heparin at a rate of 18 units/kilograms/hour without a bolus. The choice of unfractionated heparin was made due to its shorter half-life and ability to be reversed with protamine in an event of life-threatening uncontrollable hemorrhage requiring surgical intervention. The aPTT was trended based on hospital protocol to ensure therapeutic range. Inspection of the mediolateral episiotomy did not reveal any oozing, and the source of bleeding was suspected to be from the uterus. Upon discovering that the lochia had been increasing, the patient was started on 60 mg of megestrol acetate twice daily on day one with minimal improvement. The decision to start megestrol acetate was made due to the presumed subinvolution of the placenta site and limited evidence of the efficacy of megestrol acetate in treatment of subinvolution in animal models [6]. However, given the massive blood loss and the diagnosis of delayed postpartum hemorrhage, this strategy was quickly abandoned.

On hospital day 2, the patient continued to complain of progressively increasing vaginal bleeding and passage of clots. She was then started on 40 mg of oxytocin intravenously to encourage uterine contractions. Later in the evening, she reported some minimal improvement in vaginal bleeding. Over the course of 24 hours 12 peripads were soaked with blood and clots. By the end of day 2, the patient's hemoglobin and hematocrit were 9.1 g/dL and 28.1%, respectively. The oxytocin was continued for 24 hours, and bleeding was reassessed the following day. An echocardiogram was performed and demonstrated only mildly reduced right ventricular systolic function with no other abnormalities.

**Figure 3:**
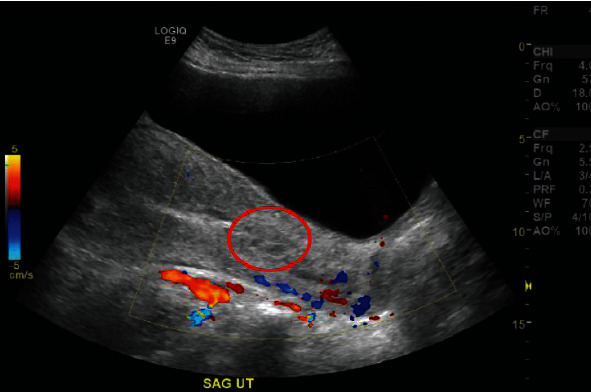
Transvaginal ultrasound of the uterus. Sagittal view of the uterus demonstrated complex echogenic area in the lower uterine segment without Doppler flow, consistent with a blood clot (encircled in red) measuring approximately 2.9 cm in diameter.

On hospital day 3, the patient continued to have excessive lochia and complained of uterine cramping. On day 3, her hemoglobin and hematocrit were 7.4 g/dL and 22.8%, respectively. Subsequently, the patient was given an intramuscular dose of 0.2 mg of methylergometrine, which was deemed to be safe in the setting of greater than 24 hours since tPA infusion. A transvaginal ultrasound was performed and revealed a complex echogenic focus without Doppler flow in the lower uterine segment consistent with a blood clot as seen in Figure 3.

On hospital day 4, she reported great improvement in her lochia overnight. However, by this point, her hemoglobin had dropped to 6.4 g/dL and her hematocrit was 19.5%. Subsequently, two units of packed red blood cells were transfused. Repeat hemoglobin was collected eight hours later and noted to be 8.4 g/dL. At this point, the patient's lochia had returned to minimal.

While the patient had been titrated to therapeutic levels of anticoagulation with unfractionated heparin, a discussion regarding the patient's lack of desire to breastfeed, and the difficulty she would encounter with the monitoring required with warfarin treatment, unfractionated heparin was discontinued, and Rivaroxaban was started. The regimen was 15 mg twice per day for 21 days then 20 mg once per day for a treatment duration of 6 months. She was advised to be aware of adverse events including black tarry stools, heavy menstrual bleeding, bleeding gums, back pain, or bowel or bladder dysfunction. She was advised that bruising and bleeding from cuts would be more pronounced.

The patient was discharged home on hospital day five and was followed up as outpatient intermittently for monitoring of ongoing treatment. The patient had a thrombophilia panel 6 weeks after her last dose of Rivaroxaban. The thrombophilia panel testing was normal.

## 3. Discussion

Pregnancy and the postpartum period are known risk factors for thromboembolic events. Factors which confer an additional risk in this population include cesarean delivery, obesity, smoking, thrombophilia, lupus, heart disease, diabetes, sickle cell disease, postpartum infection, postpartum hemorrhage, and blood transfusion [7]. However, approximately 25% of venous thromboembolic events in pregnancy are recurrent and personal history of a previous VTE confers the highest risk [7]. Researchers found that VTE was 38% higher in women aged 35 and older and 64% higher in black women [2]. Of the VTE events that occurred in pregnancy and the postpartum period, approximately 80% were deep vein thrombosis while the other 20% were pulmonary emboli [2]. Our patient denied any personal or family history of previous venous thromboembolism, and this was her first episode of pulmonary embolism. Her risk factors included obesity and pregestational diabetes in addition to being six-day postpartum. While she reported a delayed delivery of her placenta, she did not require any blood transfusion or experience postpartum hemorrhage or infection.

**Figure 4:**
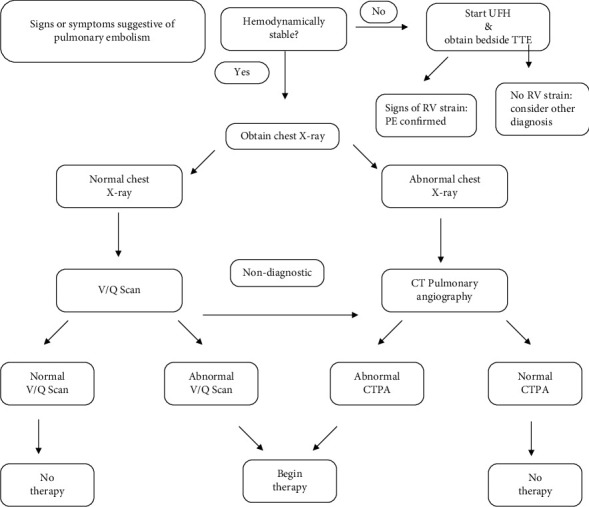
Algorithm for diagnosing pulmonary embolism in pregnancy and the postpartum period in a hemodynamically stable and unstable patient. V/Q: ventilation-perfusion; CTPA: computed tomographic pulmonary angiography; TTE: transthoracic echocardiogram; UFH: unfractionated heparin; RV: right ventricle.

Diagnosis of VTE in pregnancy and the postpartum period can be difficult due to nonspecific signs and symptoms. Specifically, the presenting symptoms of a pulmonary embolism in the postpartum period include syncope, tachycardia, chest pain, and hypotension. Oxygen desaturation is rare as a presenting sign and often associated with a large saddle embolism. Recommendations are that any woman presenting in pregnancy or the postpartum period with symptoms suggestive of pulmonary embolism should receive the appropriate imaging if hemodynamically stable [8–10]. First, a chest X-ray should be obtained. If results of the X-ray are normal, then a ventilation perfusion (V/Q) scan should be obtained next [8–10]. If chest X-ray is found to be abnormal, follow-up imaging should be a computed tomography pulmonary angiography [8–10].  Figure 4 demonstrates the basic algorithm for diagnosing pulmonary embolism in pregnancy and the postpartum period in a hemodynamically stable and unstable patient. In cases of unstable hemodynamic status, the patient should be started on therapeutic anticoagulation therapy prior to diagnostic modalities [8–10]. A bedside echocardiogram may also aid in quick diagnosis by demonstrating changes associated with right-sided heart strain (dilated right atrium/right ventricle) if computed tomography pulmonary angiography is not immediately available [8, 9]. In the present case, the patient became hemodynamically unstable, and the decision was quickly made to obtain a computed tomography angiography of the chest, which demonstrated a large saddle embolism as seen in Figures 1 and 2.

The treatment of pulmonary embolism (PE) in a nonpregnant population is based on risk stratification. The most important determinant of outcomes is hemodynamic instability. In an American Heart Association scientific statement, massive PE was defined as an acute pulmonary embolism with sustained hypotension not due to a cause other than PE, pulselessness, or persistent and profound bradycardia [11]. Patients with high risk or massive PE have been demonstrated to profoundly benefit from thrombolytic therapy in the absence of contraindications. A meta-analysis of randomized control trials, in which thrombolysis was compared with heparin in the treatment of pulmonary embolisms, found that, when used in the setting of major pulmonary embolism causing hemodynamic instability, there was a decreased rate of mortality as compared to the use of heparin alone [12]. The researchers concluded that there was benefit to the use of thrombolysis to treat hemodynamically unstable patients in the acute setting of massive pulmonary embolism with approximately a 12% bleeding risk [12]. Currently, the administration of thrombolytic therapy to individuals presenting with massive pulmonary embolisms is considered standard of care and a life-saving intervention.

While thrombolysis for the treatment of pulmonary embolism in the nonpregnant population has been demonstrated to decrease mortality, pregnancy is considered a relative contraindication [4]. Although recent surgery and bleeding are also considered relative contraindications, no mention of the postpartum period is made in the guidelines set by the American College of Chest Physicians on the use of antithrombotic therapy in VTE disease [4]. This is likely due to the lack of controlled studies and the fear of life-threatening hemorrhage in pregnancy and particularly the postpartum period. Published research is limited to multiple case studies which highlight the beneficial effects of thrombolysis in the treatment of pulmonary embolism in pregnancy. Fewer case studies exist to evaluate the use of thrombolytic treatment in the postpartum period.

A systematic review of case reports analyzed the outcomes of thrombolytics used for treatment of massive pulmonary embolism in pregnancy and the early postpartum period (limited to 48 hours after delivery) and found that there were favorable maternal outcomes [13]. However, the same study also found that there was a substantial bleeding risk that mostly pertained to the postpartum period [13]. Another systematic review of case reports analyzed the outcomes of massive and submassive pulmonary embolisms that occurred in pregnancy and up to six-week postpartum treated with thrombolysis [5]. Researchers found that in the postpartum period, when thrombolysis was used, the maternal survival rate was 86.4% but the hemorrhage rate was 58.3% when treatment occurred after delivery [5]. When stratified by the thrombolytic agent utilized, the same authors discovered that tPA had a hemorrhage risk of 5% while the hemorrhage risk after administering streptokinase was 50% when administered in the postpartum period [5]. Urokinase was also used, but less frequently [8, 13]. The most common postpartum hemorrhages were intra-abdominal bleeding in women who underwent cesarean delivery and vaginal hemorrhage in those who underwent vaginal delivery [5]. This is in contrast to a nonpregnant population with massive pulmonary embolism who receives thrombolysis, where the survival rate is estimated to be 50% to 70% and the risk of hemorrhage is approximately 12% [4, 12]. The difference in survival rate most likely reflects the younger population and better cardiovascular reserve of the pregnant and postpartum population. On the other hand, bleeding risk is significantly higher in the postpartum patient. This is due in part to recent surgery in patients after cesarean delivery, who demonstrated higher rates of intra-abdominal bleeding after administration of systemic thrombolytics [5]. Vaginal bleeding significant enough to be considered a massive postpartum hemorrhage after a vaginal delivery in the setting of systemic thrombolytics for treatment of massive pulmonary embolism can be explained by the necessity of deposition of fibrin over the placenta site as well as clots within the supplying vessels to aid in postpartum uterine involution [5].

Only case reports and case series analyzing the use of thrombolytic therapy for treatment of life-threatening embolism during pregnancy and the postpartum period exist. After reviewing the literature by searching PubMed using the words “postpartum,” “pulmonary embolism,” and “thrombolysis,” only nineteen cases could be found, derived from systematic reviews, which reported pulmonary embolism in the postpartum period treated with only thrombolytic agents in addition to therapeutic heparin [5, 8, 13, 14]. Most of these cases were either directly after delivery or prior to hospital discharge within the first two-day postpartum. Interestingly, very few case reports of pulmonary embolism utilizing thrombolytic treatment in postpartum women during the period of highest risk, as previously discussed, have been published [5, 8, 13, 14]. Only six cases have been reported of massive pulmonary embolism treated with only systemic thrombolysis after a vaginal delivery [5, 8, 13, 14]. Of those, only 2 cases occurred greater than or equal to 1-week postpartum after a vaginal delivery [5]. To the authors' knowledge, this is the only case of massive saddle pulmonary embolism occurring within the first-week postpartum after the patient was discharged home following a routine uncomplicated vaginal delivery, successfully treated exclusively with systemic thrombolysis. Furthermore, this case represents the successful use of systemic thrombolysis for the treatment and management of subsequent delayed postpartum hemorrhage with uterotonics in the setting of massive maternal pulmonary embolism.

## 4. Conclusion

This case highlights the maternal benefits of using systemic thrombolysis in the acute setting of hemodynamic decompensation secondary to a massive pulmonary embolism in the postpartum period. Given the contribution of VTE to the incidence of maternal mortality, more studies need to explore the potential beneficial effects that thrombolysis may have on decreasing maternal mortality and morbidity during pregnancy and the postpartum period. Clinicians should be aware of the potential use of thrombolysis in life-threatening cases of VTE; however, more research is needed to validate its usefulness during pregnancy and the postpartum period.

## References

[1] Petersen E. E., Davis N. L., Goodman D. (2019). Vital signs: pregnancy-related deaths, United States, 2011–2015, and strategies for prevention, 13 states, 2013–2017. *Morbidity and Mortality Weekly Report*.

[2] James A. H., Jamison M. G., Brancazio L. R., Myers E. R. (2006). Venous thromboembolism during pregnancy and the postpartum period: incidence, risk factors, and mortality. *American Journal of Obstetrics and Gynecology*.

[3] Pomp E. R., Lenselink A. M., Rosendaal F. R., Doggen C. J. M. (2008). Pregnancy, the postpartum period and prothrombotic defects: risk of venous thrombosis in the MEGA study. *Journal of Thrombosis and Haemostasis*.

[4] Kearon C., Akl E. A., Comerota A. J. (2012). Antithrombotic therapy for VTE disease: antithrombotic therapy and prevention of thrombosis, 9th ed: American College of Chest Physicians Evidence-Based Clinical Practice Guidelines. *Chest*.

[5] Martillotti G., Boehlen F., Robert-Ebadi H., Jastrow N., Righini M., Blondon M. (2017). Treatment options for severe pulmonary embolism during pregnancy and the postpartum period: a systematic review. *Journal of Thrombosis and Haemostasis*.

[6] Kumar D., Kumar A., Kumar P., Yadava C. L., PrakashYadav S. (2018). Sub-involution of placental sites (SIPS): an overview. *JournalofEntomologyandZoologyStudies*.

[7] James A. H. (2009). Venous thromboembolism in pregnancy. *Arteriosclerosis, Thrombosis, and Vascular Biology*.

[8] Rodriguez D., Jerjes-Sanchez C., Fonseca S. (2020). Thrombolysis in massive and submassive pulmonary embolism during pregnancy and the puerperium: a systematic review. *Journal of Thrombosis and Thrombolysis*.

[9] McLintock C., Brighton T., Chunilal S. (2012). Recommendations for the diagnosis and treatment of deep venous thrombosis and pulmonary embolism in pregnancy and the postpartum period. *Australian and New Zealand Journal of Obstetrics and Gynaecology*.

[10] Toglia M. R., Weg J. G. (1996). Venous thromboembolism during pregnancy. *New England Journal of Medicine*.

[11] Jaff M. R., McMurtry M. S., Archer S. L. (2011). Management of massive and submassive pulmonary embolism, iliofemoral deep vein thrombosis, and chronic thromboembolic pulmonary hypertension: a scientific statement from the American Heart Association. *Circulation*.

[12] Wan S., Quinlan D. J., Agnelli G., Eikelboom J. W. (2004). Thrombolysis compared with heparin for the initial treatment of pulmonary embolism: a meta-analysis of the randomized controlled trials. *Circulation*.

[13] Akazawa M., Nishida M. (2017). Thrombolysis with intravenous recombinant tissue plasminogen activator during early postpartum period: a review of the literature. *Acta Obstetricia et Gynecologica Scandinavica*.

[14] Jackson E., Curtis K. M., Gaffield M. E. (2011). Risk of venous thromboembolism during the postpartum period: a systematic review. *Obstetrics & Gynecology*.

